# Transcriptomic Profiling Reveals Candidate lncRNA-S100-GPCR Co-Expression Networks in Lungs of Piglets Infected with *Glaesserella parasuis*

**DOI:** 10.3390/ani16172645

**Published:** 2026-08-24

**Authors:** Jiayi Zeng, Xinqi Zeng, Xiangwei Deng, Shijia Duan, Ke Xu, Huanhuan Zhou, Hongbo Chen

**Affiliations:** 1 Laboratory of Genetic Breeding, Reproduction and Precision Livestock Farming, School of Animal Science and Nutritional Engineering, Wuhan Polytechnic University, Wuhan 430023, China; 2 Hubei Provincial Center of Technology Innovation for Domestic Animal Breeding, Wuhan Polytechnic University, Wuhan 430023, China

**Keywords:** *Glaesserella parasuis*, GPCR, inflammation, lncRNA, pig, S100 family, transcriptomics

## Abstract

*Glaesserella parasuis* (*G. parasuis*) is a major respiratory pathogen in piglets, but the regulatory mechanisms underlying its induced pulmonary inflammation remain poorly understood. In this study, whole-transcriptome sequencing was carried out on lung tissues from colostrum-deprived piglets with mild and severe serotype 5 *G. parasuis* infection and healthy controls. Differential expression (DE) analysis revealed 299 nominally DE mRNAs and 408 nominally DE lncRNAs in the mild group, increasing to 625 and 1193, respectively, in the severe group. Ingenuity Pathway Analysis identified the S100 family signaling pathway as a core inflammatory module predicted to be activated across both infection grades, with its transcriptional involvement expanding from 8 genes in mild infection to 42 genes in severe infection. Notably, G-protein-coupled receptors (GPCRs) accounted for nearly half (19/42) of the S100-associated DE genes in severe infection, covering multiple functional categories including chemokine receptors, lipid mediator receptors, and metabotropic receptors, suggesting systemic activation of the GPCR family in severe inflammation. Weighted gene co-expression network analysis identified multiple lncRNA candidates, among which two—*LOC110256217* and *LOC110259349*—showed severity-associated connectivity patterns and were selected for further validation. Following *G. parasuis* infection, time-series RT-qPCR in 3D4/21 cells confirmed their co-expression with corresponding mRNAs and revealed distinct temporal patterns, suggesting their potential differential involvement at early and late stages of the inflammatory response. Collectively, these findings identify a putative lncRNA-S100-GPCR-associated inflammatory module linked to pulmonary inflammation in *G. parasuis* infection, providing a transcriptomic resource and candidate lncRNA-mRNA pairs for further functional studies and investigation into host resilience. Given the limited sample size (*n* = 3 per group), these findings should be considered exploratory and warrant validation in larger cohorts.

## Simple Summary

*Glaesserella parasuis* (*G. parasuis*) is a major bacterial pathogen that causes severe respiratory disease in piglets, leading to substantial economic losses worldwide. However, the molecular mechanisms underlying lung inflammation following infection remain incompletely understood. In this study, whole-transcriptome sequencing was conducted on lung tissues from colostrum-deprived piglets with mild and severe serotype 5 *G. parasuis* infection and healthy controls. The number of differentially expressed genes and long non-coding RNAs increased progressively with disease severity. One prominent signaling pathway, the S100 family, was predicted to be activated in both mild and severe infections, but its involvement was markedly broader in severe cases, involving more molecules such as GPCRs and matrix metalloproteinases. Two long non-coding RNAs—*LOC110256217* and *LOC110259349*—that are co-expressed with core genes in this pathway at different stages of infection were identified via co-expression network analysis. These findings suggest a putative lncRNA-S100-GPCR-associated inflammatory module in pulmonary inflammation induced by *G. parasuis*. This work provides valuable resources for understanding the molecular basis of lung inflammation to advance investigations into host-bacterial interactions.

## 1. Introduction

*Glaesserella parasuis* (*G. parasuis*) is a Gram-negative short rod-shaped bacterium that serves as an early colonizer of the upper respiratory tract in pigs and is commonly present in healthy herds [1]. When host immunity declines or is under stress conditions, pathogenic strains can breach the respiratory mucosal barrier, spread to the lungs and cause pneumonia, and may even invade systemic tissues and organs. This can lead to typical lesions such as polyserositis, meningitis, and polyarthritis, which are collectively referred to as Glässer’s disease [2]. *G. parasuis* is classified into 15 serotypes [3,4], among which serotypes 4, 5, 12, and 13 are prevalent in China [5], posing a serious threat to the pig industry both in China and worldwide [6,7].

Studies have shown that *G. parasuis* employs multiple virulence factors for colonization, immune evasion, and tissue invasion in pigs [8,9]. These virulence factors mainly include lipooligosaccharide (LOS) [10], outer membrane proteins (such as OmpP2 and OmpP5) [11], capsular polysaccharide [12], cytolethal distending toxin (CDT) [13], and pili (such as PilA) [14]. During infection, these virulence factors, including LOS [15] and the newly identified HbpA [16], activate host inflammatory signaling pathways via TLR2/TLR4, inducing the release of large amounts of pro-inflammatory cytokines, such as *IL-6* and *TNF-α*, and leading to immunopathological damage in the lungs. In addition, *G. parasuis* can also induce pyroptosis in alveolar macrophages through the endoplasmic reticulum stress PERK/eIF2α/ATF4 axis [17] and activate the NLRP3 inflammasome signaling pathway [18], further exacerbating the inflammatory response. Recent studies have also found that *RHOA* acts as a host-dependent factor during *G. parasuis* infection of LLC-PK1 cells in vitro, and its knockdown significantly reduces bacterial adhesion and invasion [19]. These studies provide important theoretical foundations for anti-inflammatory therapy and vaccine development.

Long non-coding RNAs (lncRNAs) are a class of non-coding RNA molecules longer than 200 nucleotides that participate in gene expression regulation through chromatin modification, transcription, and epigenetic regulation [20]. Recent studies have shown that lncRNAs play important regulatory roles in inflammatory responses and bacterial infections [21,22], with some lncRNAs modulating the production of inflammatory cytokines by regulating the activity of inflammatory signaling pathways such as NF-κB [23,24,25]. In porcine infection models, lncRNAs have also been confirmed to participate in the regulation of inflammation induced by pathogens such as porcine reproductive and respiratory syndrome virus (PRRSV) [26], *Clostridium perfringens* [27], porcine epidemic diarrhea virus (PEDV) [28], and swine influenza virus (SIV) [29], suggesting that lncRNAs may serve as potential intervention targets for inflammation induced by bacterial pathogens in pigs. In *G. parasuis* infection, previous studies have analyzed lncRNA expression profiles using in vitro models such as 3D4/21 cells [30] and porcine aortic vascular endothelial cells (PAVECs) [31], revealing that *G. parasuis* infection induces significant changes in host lncRNA expression profiles. In recent years, the functions of some lncRNAs have been gradually elucidated. For example, lncRNA-MEG3 participates in *G. parasuis*-induced inflammatory responses and apoptosis through the miR-210/TLR4 axis [32], and can also promote apoptosis by sponging ssc-miR-135 to upregulate *CASP8* expression [33]. However, these studies are limited to in vitro cell models, and the whole-transcriptome expression profiles and lncRNA-mRNA co-expression networks in lung tissue inflammation during *G. parasuis* infection remain unclear.

In this study, whole-transcriptome sequencing was performed on lung tissues from healthy controls and infected piglets using a previously established *G. parasuis* infection model. LncRNA-mRNA co-expression networks were constructed, and correlated transcript pairs were dynamically validated in vitro using a 3D4/21 cell infection model. These analyses provide candidate molecular correlates for elucidating the inflammatory mechanisms induced by *G. parasuis*.

## 2. Materials and Methods

### 2.1. Animal Model Establishment and Tissue Sample Collection

The colostrum-deprived (CD) piglet model infected with *Glaesserella parasuis* serotype 5 strain SH0165 was established based on our previously published research [1,34]. All animal operations were approved by the Animal Care and Use Committee of Wuhan Polytechnical University (WPU202108002, 1 August 2021). Briefly, nine Duroc × Landrace × Yorkshire newborn piglets were artificially fed with a milk replacer without sow colostrum and received no vaccines until weaning at 21 days of age. Pathogen screening for *G. parasuis*, *Actinobacillus pleuropneumoniae*, *Streptococcus suis*, pseudorabies virus, classical swine fever virus, and PRRSV was conducted before infection. At 30 days of age, six piglets were intratracheally challenged with 2 × 10^8^ CFU *G. parasuis*, while three uninfected piglets served as the control group. Clinical manifestations of infected piglets were monitored continuously within 48 h post-infection (hpi). At 48 hpi, all piglets were scored using a six-item clinical grading system (behavior, respiration, lameness/joint swelling, neurological signs, cough, feed intake), with each indicator scored 0 (normal), 1 (mild abnormality) or 2 (severe abnormality) as described in Table S1 and Ref. [34]. Based on total clinical scores, the six infected piglets were divided into mild (total scores 1–3, *n* = 3) and severe (total scores 6–10, *n* = 3) subgroups; all control piglets scored 0. All piglets were euthanized at 48 hpi, and lung tissues were collected. Samples were divided into two parts: one fixed in 4% paraformaldehyde for histopathological observation, the other snap-frozen in liquid nitrogen and stored at −80 °C for transcriptome sequencing and RT-qPCR detection.

### 2.2. Paraffin Section Preparation and Hematoxylin–Eosin Staining

Lung tissues were fixed in 4% paraformaldehyde for at least 24 h post-sampling. After fixation, tissues were subjected to automatic dehydration and wax infiltration via a tissue processor, serially treated with graded ethanol, absolute ethanol, ethanol–xylene mixture and pure xylene prior to wax impregnation. For embedding, infiltrated tissues were placed into embedding cassettes filled with molten paraffin according to the required cutting surfaces for solidification, followed by trimming of excess paraffin around tissue blocks. The trimmed blocks were sliced into thin sections. Obtained slices were floated in warm water for flattening, mounted onto glass slides and oven-dried, then stored at room temperature for subsequent staining. After baking, sections underwent dewaxing and rehydration procedures, followed by hematoxylin–eosin staining, differentiation and blue reversal. Stained slices were further dehydrated and cleared, and finally mounted with neutral balsam. All stained histological sections were observed under a light microscope (Nexcope, Ningbo, China), and images were captured using ImageView Software (x64, 4.10.17350.20200621).

### 2.3. Quantitative Real-Time PCR (RT-qPCR)

Total RNA extracted from lung tissues was reverse-transcribed into cDNA using the PrimeScript™ RT reagent Kit with gDNA Eraser (TaKaRa, Osaka, Japan). The relative mRNA expression levels of target genes were quantified via TB Green^®^ Premix Ex Taq™ II (TaKaRa, Osaka, Japan) following the manufacturer’s protocols. RT-qPCR reactions were carried out on a QuantStudio™ 1 Plus real-time fluorescence quantitative PCR instrument (Thermo Fisher Scientific, Waltham, MA, USA). The 10 μL amplification system was composed of 5 μL TB Green II, 3.6 μL RNase-free water, 1 μL cDNA template, and 0.2 μL of each forward and reverse primer. Amplification procedures were performed as recommended by the kit instructions, including initial denaturation at 95 °C and 40 amplification cycles (95 °C for 10 s, gene-specific annealing for 30 s). Three technical replicates were set for each sample, and *GAPDH* was selected as the reference gene for normalization. The relative gene expression data were calculated by the 2^−ΔΔCt^ method as previously described [35]. All primer pairs were validated via melt-curve analysis for single-product amplification, and *GAPDH* showed consistent Ct values across all samples. All primer sequences used for RT-qPCR analysis are listed in Table S2.

### 2.4. RNA Sequencing and Transcriptomic Analysis

Total RNA was extracted from lung tissues using TRIzol reagent (TaKaRa, Osaka, Japan), with three biological replicates per group. RNA quality and integrity were assessed via NanoDrop spectrophotometer (Thermo Fisher Scientific, Waltham, MA, USA), Qubit 4.0, and Agilent 2100. After rRNA depletion, strand-specific transcriptome libraries were constructed and sequenced on the DNBSEQ-T7 platform (PE150). Raw reads were filtered using fastp (v0.21.0) and aligned to the porcine reference genome Sscrofa11.1 with HISAT2 (v2.1.0). Gene expression levels were quantified by featureCounts (v2.0.0) and normalized to fragments per kilobase of transcript per million mapped fragments (FPKM). Differential expression (DE) analysis of mRNAs was performed using DESeq2 (v1.30.1), with |log_2_FoldChange| ≥ 1 and raw *p* < 0.05 as screening cutoffs. As no genes survived multiple-testing correction (FDR), these transcripts were considered nominally differentially expressed and were carried forward for exploratory downstream analyses. Functional annotation of these transcripts was performed using Ingenuity Pathway Analysis (IPA, QIAGEN, release 2025), including canonical pathway enrichment, disease and function analysis. The default Ingenuity Knowledge Base served as the gene-universe background; porcine gene symbols were internally mapped to human orthologs within IPA. Canonical pathway enrichment *p*-values were calculated by right-tailed Fisher’s exact test. Pathway enrichment significance was determined based on the IPA overlap *p*-value (−log(*p*-value)). The activation Z-score was employed exclusively for inferring pathway activation or inhibition, rather than for assessing enrichment significance.

### 2.5. Identification and Differential Expression of lncRNAs

Transcripts were assembled de novo using StringTie (v1.2.4). Candidate lncRNAs were filtered based on length ≥ 200 bp, exon number ≥ 2, class code (x: antisense; u: intergenic; i: intronic), and coverage > 3 in at least one sample. Four coding potential tools (CPC, CNCI, Pfam, PLEK) were jointly applied, and transcripts predicted as non-coding by all four tools were retained as high-confidence novel lncRNAs. Known lncRNAs were extracted from the porcine reference genome annotation file (GFF). The novel and known lncRNAs were combined to generate the complete lncRNA expression dataset. Transcripts with FPKM > 0.01 were considered expressed. Differential expression of lncRNAs was evaluated with DESeq2 (v1.30.1) using the same thresholds as mRNAs.

### 2.6. LncRNA-mRNA Co-Expression Network Construction

To identify co-expressed mRNAs of DE lncRNAs, weighted gene co-expression network analysis (WGCNA, v1.72-5) was performed using the FPKM expression profiles of DE lncRNAs and DE mRNAs from all samples. A soft-thresholding power of 7 was applied to ensure a scale-free network. Pearson correlation coefficients were calculated for each lncRNA-mRNA pair, and co-expression pairs with a weight value > 0.6 were retained as significant co-expression links. Notably, given the limited sample size (*n* = 9), this WGCNA analysis is only used for exploratory screening of candidate correlated transcript pairs, rather than establishing definitive, stable global regulatory modules. The resulting co-expression networks were visualized as heatmaps using the R package pheatmap (v1.0.12) and Cytoscape (v3.10.3) subnetworks. The proposed model was illustrated using Figdraw (https://www.figdraw.com/#/).

### 2.7. Bacterial Strain and Cell Culture

*G. parasuis* strain SH0165 (serotype 5) was cultured at 37 °C in tryptic soy broth (TSB; BD Biosciences, Franklin Lakes, NJ, USA) or on tryptic soy agar (TSA; BD Biosciences) supplemented with 10% fetal bovine serum (FBS; Gibco, Thermo Fisher Scientific, Waltham, MA, USA) and 10 µg/mL β-nicotinamide adenine dinucleotide (NAD; Beyotime Biotechnology, Shanghai, China). For preparation of bacterial inoculum, a single colony was inoculated into TSB and grown at 37 °C with orbital shaking at 220 rpm until the optical density at 600 nm (OD_600_) reached 0.6–0.7.

Porcine 3D4/21 cells were cultured in RPMI 1640 medium (Gibco, Thermo Fisher Scientific, USA) supplemented with 10% FBS (Cell-Box, Shanghai, China) and 1% Penicillin Streptomycin (Gibco, Thermo Fisher Scientific, Waltham, MA, USA). Cells were incubated at 37 °C in a humidified atmosphere (Thermo Fisher Scientific, USA) with 5% CO_2_. For infection, cells were cultured in RPMI 1640 with 2% FBS without antibiotics, then infected with the bacteria at a multiplicity of infection (MOI) of 10 and harvested at the indicated time points.

### 2.8. Cell Viability Assay

Cell viability was determined using the CellTiter-Lumi Luminescent Cell Viability Assay Kit (Beyotime Biotechnology, Shanghai, China). Following infection, 3D4/21 cells were harvested by trypsinization with 0.25% EDTA-trypsin (Gibco, Thermo Fisher Scientific, Waltham, MA, USA), counted, and replated into 96-well plates at uniform densities. Subsequently, a volume of CellTiter-Lumi reagent equivalent to the culture medium was added to each well. Plates were gently agitated on an orbital shaker for 2 min and then incubated at room temperature for 10 min with protection from light. Luminescent signals were recorded using an LB960 XS3 luminometer (Berthold Technologies, Bad Wildbad, Germany). Each experimental group contained three biological replicates, and each replicate was measured in triplicate technical repeats.

### 2.9. Statistical Analysis

All data are presented as mean ± standard deviation (SD). Statistical analyses were performed using GraphPad Prism 10.6.0 (GraphPad Software, Boston, MA, USA). For multiple group comparisons, one-way ANOVA followed by Tukey’s or Dunnett’s post-hoc test was used as appropriate. For two-group comparisons, unpaired Student’s *t*-test with Welch’s correction was applied. A *p*-value < 0.05 was considered statistically significant.

## 3. Results

### 3.1. G. parasuis Infection Triggers Graded Pulmonary Inflammatory Lesions in CD Piglets

**Figure 1:**
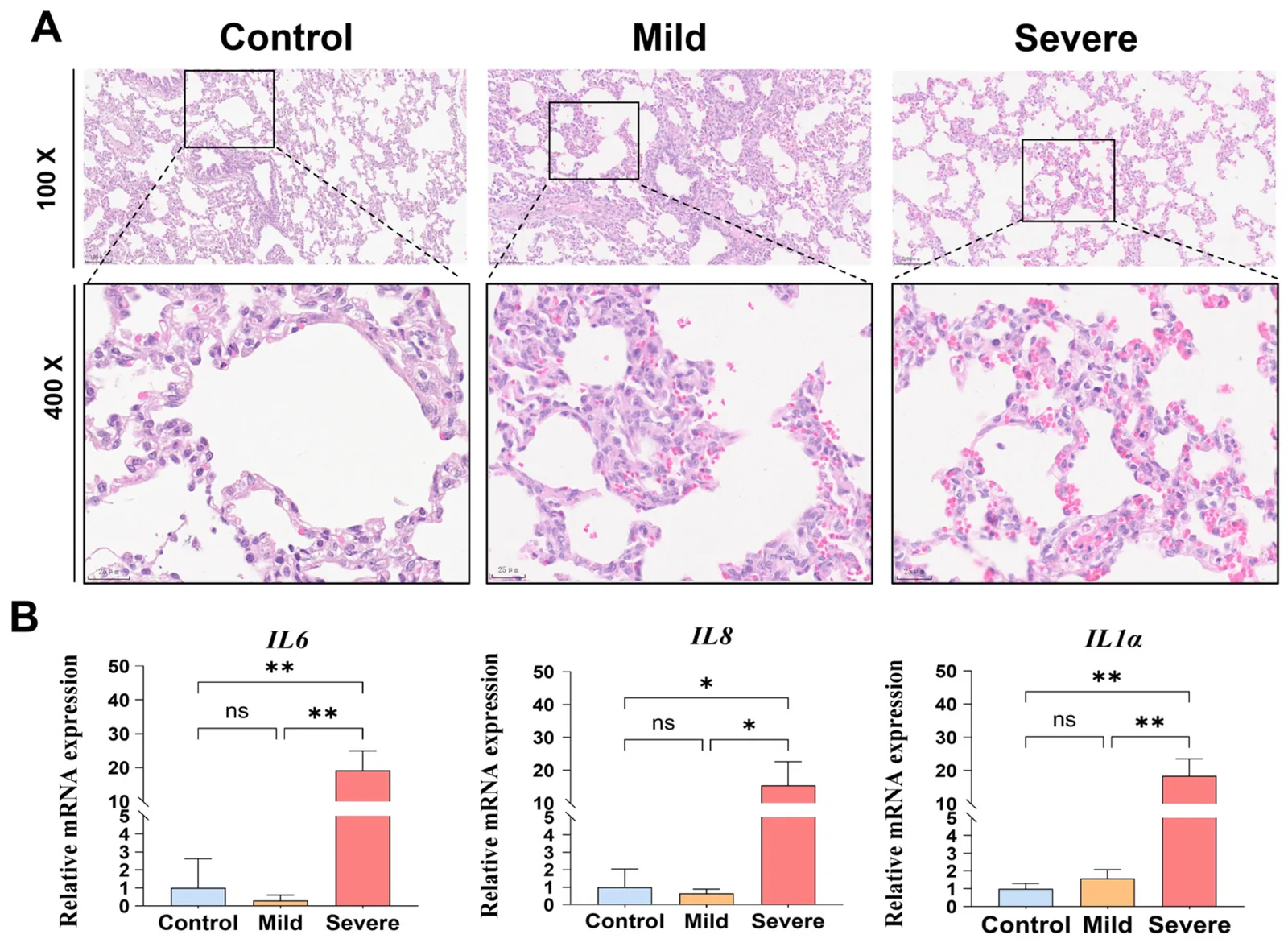
*G. parasuis* infection induces graded pulmonary inflammatory responses in colostrum-deprived piglets. (**A**) Representative HE-stained pathological images of lung tissues from control, mild and severe groups. Low magnification: 100×; scale bar = 100 μm; high magnification: 400×; scale bar = 25 μm. (**B**) Relative mRNA expression of pro-inflammatory cytokines *IL1α*, *IL6*, and *IL8* were determined by RT-qPCR. Data are presented as mean ± SD, and statistical analysis was performed using one-way ANOVA followed by Tukey’s post-hoc test. * *p* < 0.05, ** *p* < 0.01; ns, *p* ≥ 0.05.

Based on our previously constructed CD piglet infection model of *G. parasuis*, three cohorts (control, mild, severe) were defined via standardized clinical scoring. Subsequently, histopathological observation via HE staining was performed on lung tissues from each group to characterize tissue morphological changes. Compared with the uninfected control group, piglets in the mild group displayed mild thickening of alveolar septa and limited inflammatory cell infiltration. In stark contrast, the severe group exhibited extensive inflammatory cell infiltration accompanied by prominent erythrocyte extravasation, indicating noticeably aggravated lung tissue damage (Figure 1A).

To further quantify the gradient of pulmonary inflammatory response following *G. parasuis* challenge, RT-qPCR was adopted to detect the mRNA expression of classic pro-inflammatory cytokines *IL1α*, *IL6* and *IL8* in lung tissues of all groups. No statistically significant differences in these cytokine transcripts were observed between the control and mild groups. However, the expression levels of all three pro-inflammatory genes were significantly upregulated in the severe group compared with the other two cohorts (Figure 1B). Taken together, histological and molecular evidence verified that the clinical severity of *G. parasuis*-infected piglets was positively correlated with the magnitude of local pulmonary inflammatory damage, and pulmonary inflammation exhibited distinct graded progression characteristics after bacterial infection.

### 3.2. DE mRNAs Are Enriched in Disease-Related Canonical Pathways, with S100 Family Signaling as a Consistently Activated Signature

**Figure 2:**
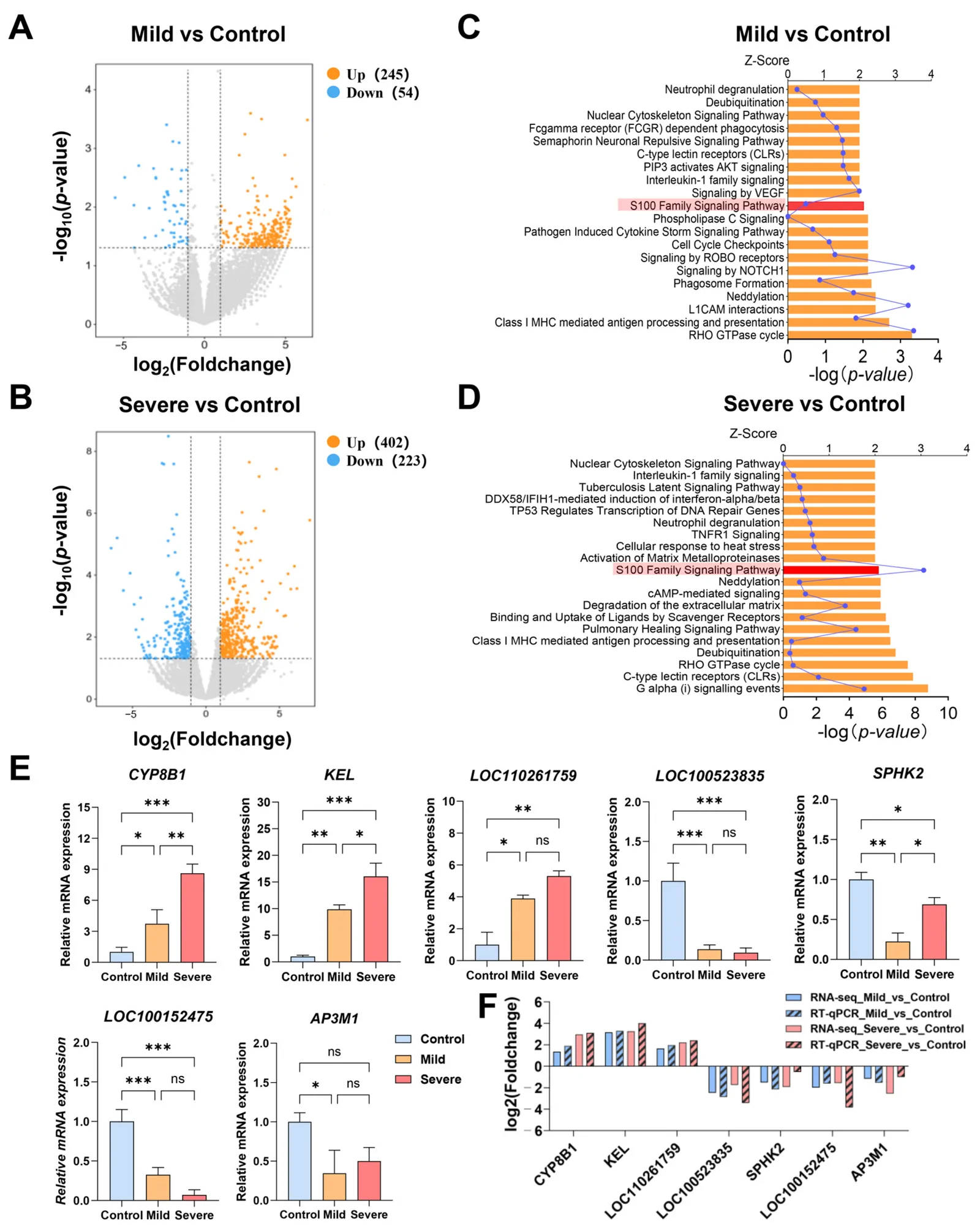
Identification of DE mRNAs and IPA of disease-related canonical pathways. (**A**,**B**) Volcano plots of differentially expressed mRNAs in (**A**) Mild vs. Control and (**B**) Severe vs. Control groups. Up- and down-regulated genes (|log_2_FC| ≥ 1, *p* < 0.05) are shown in orange and blue, respectively, whereas non-significant genes are shown in grey. Dotted lines indicate the thresholds of |log_2_FC| = 1 and *p* = 0.05, with numbers indicated. *n* = 3. (**C**,**D**) IPA of nominally DE mRNAs in (**C**) Mild vs. Control and (**D**) Severe vs. Control groups. The top 20 pathways biologically relevant to pulmonary infection, inflammation and tissue injury are shown. Blue dots indicate − log(*p*-value) for pathway enrichment, and orange bars represent the Z-score for predicted pathway activation or inhibition status. (**E**) RT-qPCR validation of seven randomly selected DE mRNAs. Bar graphs show relative mRNA expression (mean ± SD, *n* = 3), normalized to *GAPDH* and presented as fold change relative to the control group. (**F**) Scatter plot comparing log_2_(Fold Change) values from RNA-seq and RT-qPCR. Statistical significance was determined by one-way ANOVA with Tukey’s post-hoc test. * *p* < 0.05, ** *p* < 0.01, *** *p* < 0.001; ns, *p* ≥ 0.05.

To identify shared transcriptomic drivers of pulmonary inflammation induced by *G. parasuis*, RNA-seq was performed on lung tissues from control, mild and severe piglets to profile DE mRNAs. Table S3 presents RNA-seq QC and mapping statistics for all lung samples. PCA clearly separated control and severe samples into distinct clusters, with mild piglets falling in the intermediate transcriptional space (Figure S1), revealing graded lung transcriptional remodeling correlated with infection severity. Differential expression analysis identified 299 DE mRNAs (245 upregulated, 54 downregulated) in the Mild vs. Control comparison and 625 DE mRNAs (402 upregulated, 223 downregulated) in the Severe vs. Control comparison (Figure 2A,B; Tables S4 and S5). IPA was further applied to characterize canonical signaling cascades enriched in DE mRNA datasets. All enriched Ingenuity canonical pathways are listed in Tables S6 and S7, and Disease and Function enrichment outputs are available in Tables S8 and S9. Among these, the top 20 pathways biologically relevant to pulmonary infection, inflammation and tissue injury were selected for visualization (Figure 2C,D). Core innate immune cascades shared by both infection grades included neutrophil degranulation, interleukin-1 family signaling, C-type lectin receptor (CLR) signaling and class I MHC antigen processing and presentation. Mild-specific pathways covered Fcγ receptor-dependent phagocytosis, VEGF-mediated angiogenic signaling and NOTCH1 signal transduction, while severe infection uniquely enriched DDX58/IFIH1-driven interferon antiviral responses, matrix metalloproteinase-mediated extracellular matrix degradation and pulmonary wound-healing pathways.

Consistent with the canonical pathway findings, IPA Diseases and Functions enrichment revealed that mild samples were predominantly enriched for cell migration, cytoskeleton remodeling, and localized epithelial injury, whereas severe samples showed stronger enrichment of anti-infective responses, immune cell differentiation, leukocyte adhesion, lesion angiogenesis, and tissue remodeling (Figure S2; Tables S8 and S9). RT-qPCR validation of seven randomly selected DE mRNAs confirmed the RNA-seq expression trends (Pearson’s *r* = 0.926, *p* < 0.0001), with most comparisons statistically significant (Figure 2E,F).

Notably, the S100 Family Signaling pathway was predicted to be activated across infected piglets of all lesion grades. While comparable pathway activation was predicted in both groups, the magnitude of transcriptional perturbation and the number of enriched genes were markedly elevated under severe infection. Distinct DE gene repertoires contributed to this shared inflammatory cascade between mild and severe samples. Within the S100 signaling module, only 8 genes were enriched in mild infection, including *GHSR*, *ADGRG6*, and *CHRM3*, while an expanded set of 42 pathway members were detected in severe lesions, such as *PTGDR2*, *LTB4R2*, and *MMP1*. The IPA molecular interaction network of this cascade is presented in Figure S3.

### 3.3. Identification and Characterization of DE lncRNAs in the Lungs of CD Piglets Infected with G. parasuis

**Figure 3:**
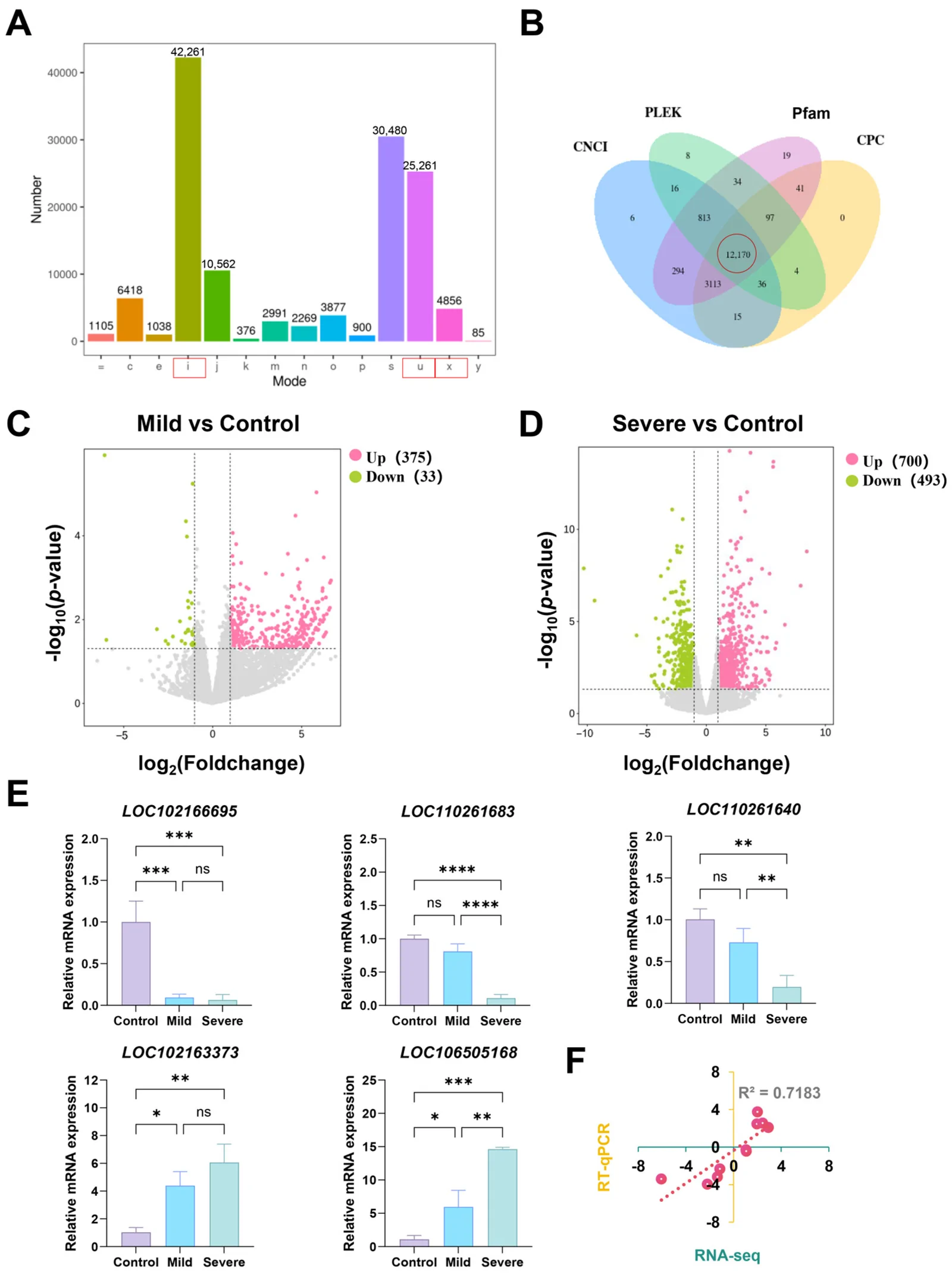
Identification of DE lncRNAs in the lungs of *G. parasuis*-infected CD piglets. (**A**) Class_code distribution of assembled transcripts. The red box highlights three classes of lncRNA transcripts, namely x (antisense), u (intergenic), and i (intronic). (**B**) Venn diagram showing novel lncRNAs identified by four prediction tools (CPC, CNCI, Pfam, PLEK). The red circle marks the intersection set. (**C**,**D**) Volcano plots of DE lncRNAs in mild vs. control (**C**) and severe vs. control (**D**) groups (|log_2_FoldChange| ≥ 1, and *p* < 0.05). Non-significant lncRNAs are shown in grey, and dashed lines represent the threshold values of |log_2_FC| = 1 and *p* = 0.05. (**E**) RT-qPCR validation of randomly selected DE lncRNAs. Data are mean ± SD (*n* = 3), normalized to *GAPDH*; one-way ANOVA with Tukey’s test. (**F**) Pearson correlation between RT-qPCR and RNA-seq data (log_2_FoldChange). * *p* < 0.05, ** *p* < 0.01, *** *p* < 0.001, **** *p* < 0.0001; ns, *p* ≥ 0.05.

To explore the potential involvement of lncRNAs in pulmonary inflammation, the lncRNA expression landscape in infected piglets was systematically characterized. Following transcript assembly, class_code analysis showed that types “x” (antisense), “u” (intergenic), and “i” (intronic) accounted for the majority of non-coding transcripts (Figure 3A). Using four prediction tools (CPC, CNCI, Pfam, and PLEK), 12,170 high-confidence novel lncRNAs were identified at their intersection (Figure 3B), which, together with 5605 known lncRNAs from genome annotation, constituted the lncRNA analysis library. Characterization of the identified lncRNAs is summarized in Figure S4A–F. Notably, PCA revealed a graded separation among the three groups, with the mild group positioned between control and severe (Figure S4F), consistent with the mRNA expression patterns and pathological progression.

Differential expression analysis revealed 408 DE lncRNAs in the mild group (375 upregulated, 33 downregulated) and 1193 DE lncRNAs in the severe group (700 upregulated, 493 downregulated) relative to controls (Figure 3C,D; Tables S10 and S11). The number of DE lncRNAs increased progressively with the severity of pulmonary inflammation, paralleling the graded pathological damage and mRNA dysregulation observed above. RT-qPCR validation of five randomly selected DE lncRNAs confirmed the RNA-seq results (Figure 3E), with a strong correlation between the two platforms (*r* = 0.8475, *p* < 0.0001; Figure 3F).

### 3.4. Co-Expression Network Analysis of DE lncRNAs and Co-Expressed mRNAs

**Figure 4:**
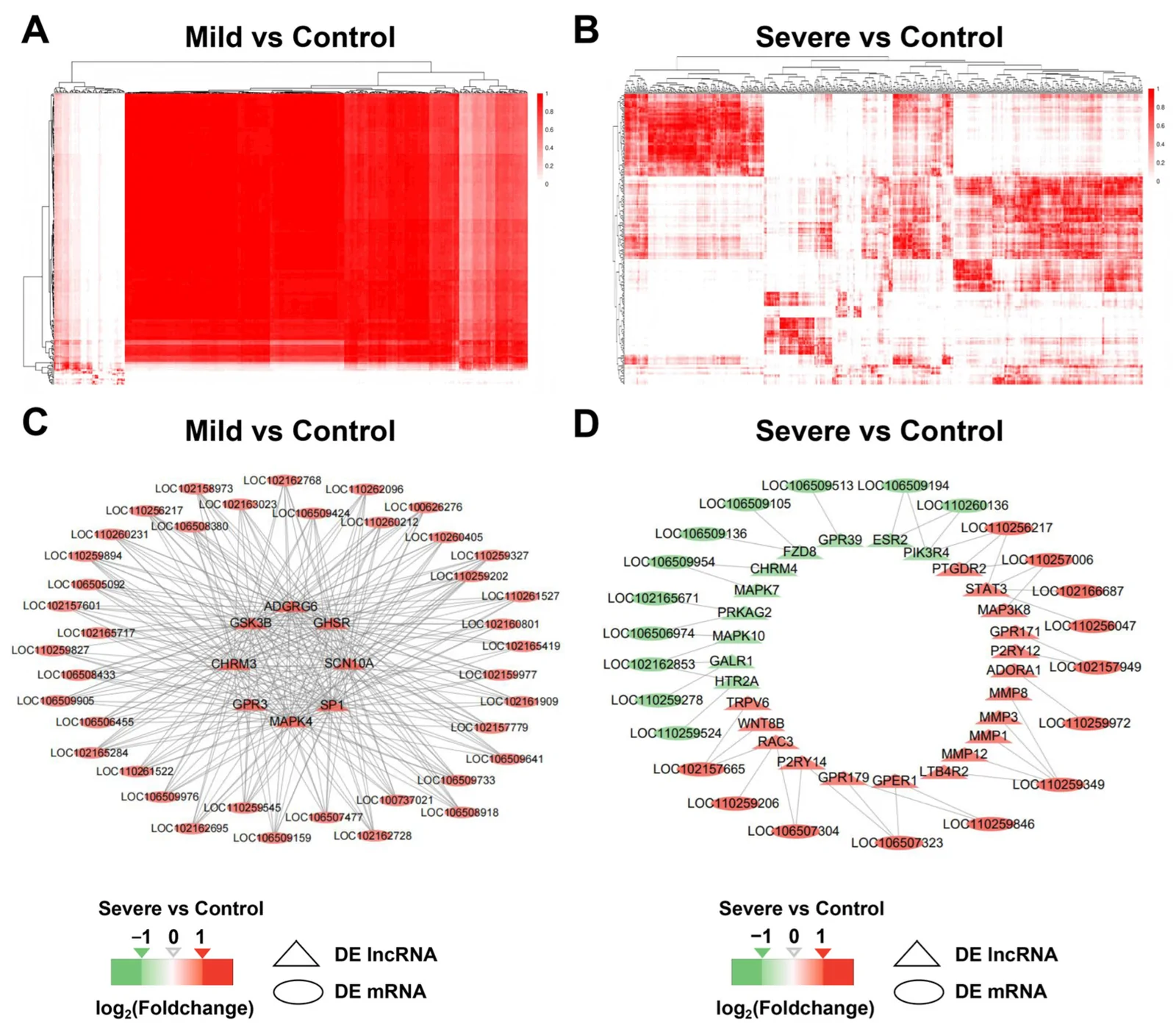
Co-expression networks of DE lncRNAs and co-expressed mRNAs. (**A**,**B**) Global co-expression heatmaps of all DE lncRNA–DE mRNA pairs in the Mild vs. Control (**A**) and Severe vs. Control (**B**) comparisons (soft-thresholding power = 7). (**C**,**D**) Cytoscape subnetworks of nominally DE lncRNAs and S100 pathway-related nominally DE mRNAs, extracted from significant co-expression pairs (weight > 0.6). Triangles represent lncRNAs, circles represent mRNAs. Node color indicates log_2_FC (red: upregulated, green: downregulated).

To explore the potential roles of DE lncRNAs in *G. parasuis*-induced pulmonary inflammation, weighted gene co-expression networks were constructed for Mild vs. Control and Severe vs. Control comparisons. Global TOM clustering of DE lncRNAs and their co-expressed mRNAs (Figure S5) revealed that mild infection was associated with weak overall transcriptional coherence and indistinct module partitioning, whereas severe infection exhibited elevated within-module co-expression strength and clearer modular segregation. The analysis then focused on pairwise co-expression weights across all DE lncRNA–DE mRNA pairs (Figure 4A,B). The mild group showed generally weak co-expression across most transcript pairs. By contrast, the severe group displayed stronger and more concentrated co-expression signals. Pairs with co-expression weight > 0.6 were defined as significant co-expressed pairs for subsequent network visualization, and all qualified pairs are listed in Tables S12 and S13.

Given that the S100 family signaling pathway was identified as a core inflammatory signature in the mRNA transcriptome analysis (Section 3.2), subgroup-specific subnetworks for S100 pathway-related transcripts (Figure 4C,D) were further constructed. In the mild group, a limited set of S100-related mRNAs was paired with abundant upregulated DE lncRNAs, forming tightly clustered hub interactions. In the severe group, a larger pool of S100-associated mRNAs was paired with fewer total DE lncRNAs, with both upregulated and downregulated lncRNAs participating in dispersed co-expression patterns. Notably, all DE lncRNAs within the S100-associated subnetworks were annotated known lncRNAs; no novel lncRNAs were identified in this co-expression subset. These screened lncRNA–mRNA pairs provide prioritized candidates for follow-up functional validation.

Among these S100-associated pairs, two annotated known lncRNAs (*LOC110256217* and *LOC110259349*) displayed connectivity patterns correlated with infection severity. *LOC110256217* was paired with 8 S100-related genes in the mild group (*ADGRG6*, *GSK3B*, *GHSR*, *CHRM3*, *SCN10A*, *GPR3*, *SP1*, and *MAPK4*), but its connectivity narrowed to 3 genes in the severe group (*PTGDR2*, *PIK3R4*, and *STAT3*). *LOC110259349*, by contrast, appeared exclusively in the severe network, where it was co-expressed with 5 effector genes (*MMP1/3/8/12* and *LTB4R2*). These distinct interaction shifts suggest that the two lncRNAs may be differentially associated with different disease stages, and they were therefore selected as representatives for subsequent temporal expression validation. The genomic features of *LOC110256217* and *LOC110259349* are summarized in Table S14.

### 3.5. In Vitro Infection Model in 3D4/21 Cells Validates lncRNA-mRNA Co-Expression Within the S100 Pathway

**Figure 5:**
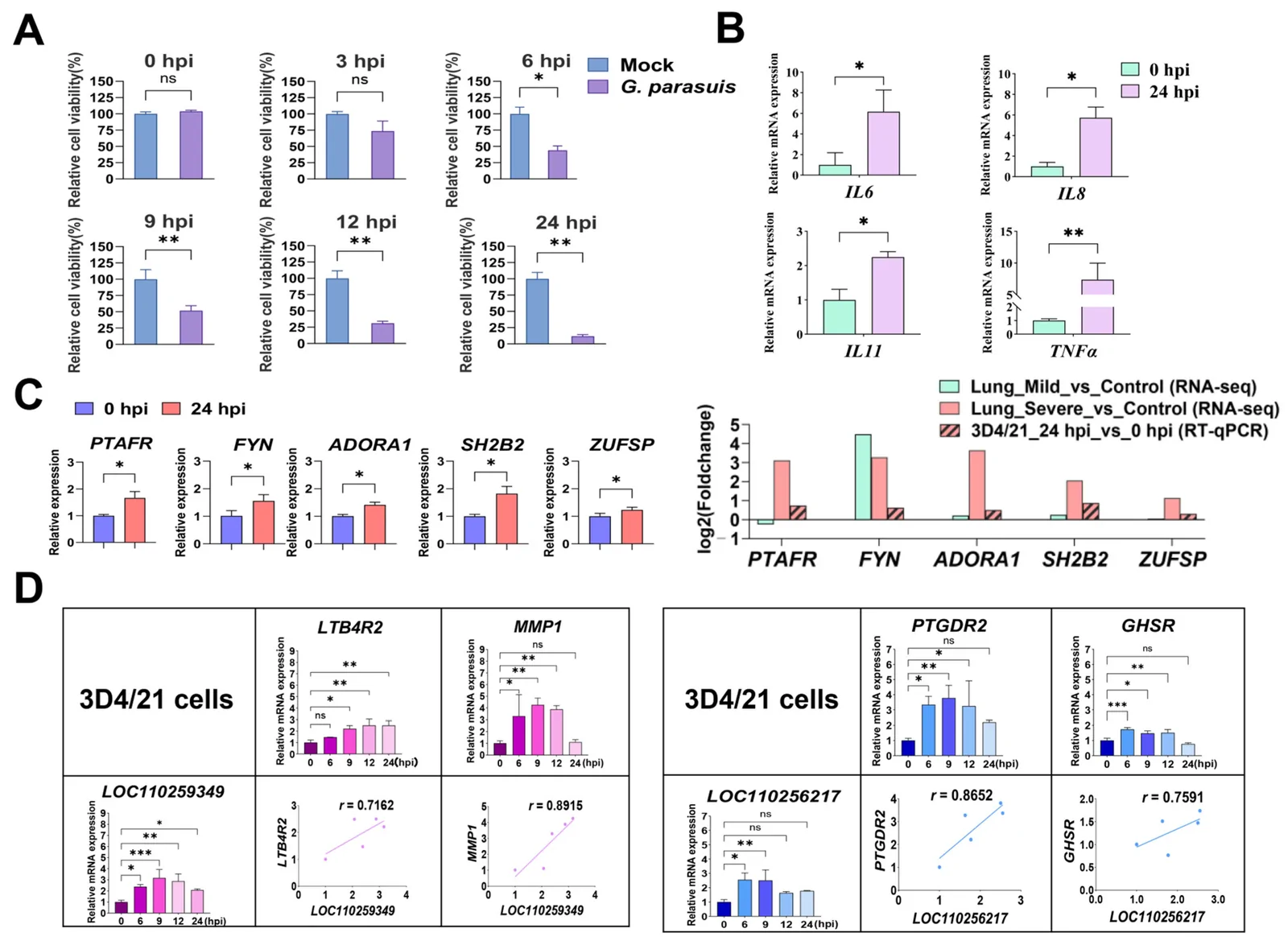
Establishment of an in vitro *G. parasuis* infection model in 3D4/21 cells and validation of lncRNA-mRNA co-expression relationships involved in the S100 pathway. (**A**) Relative cell viability of 3D4/21 cells at 0, 3, 6, 9, 12, and 24 h post-infection (hpi) with *G. parasuis*. (**B**) RT-qPCR quantification of pro-inflammatory cytokine (*IL6*, *IL8*, *IL11*, *TNF*α) mRNA expression at 0 hpi and 24 hpi. Expression levels were normalized to *GAPDH*. (**C**) **Left panel**: RT-qPCR detection of gene expression in 3D4/21 cells at 24 hpi relative to 0 hpi. **Right panel**: Comparison of log_2_(fold change) values between cellular RT-qPCR and lung RNA-seq data. (**D**) Time-series RT-qPCR validation of nominally DE lncRNA–DE mRNA co-expression in the S100 pathway at 0, 6, 9, 12, and 24 hpi. Pearson correlation coefficients (*r*) for all tested pairs are shown (*p* < 0.0001). Statistical significance was determined by unpaired Student’s *t*-test with Welch’s correction (**A**–**C**) or one-way ANOVA followed by Dunnett’s multiple comparisons test versus 0 hpi (**D**). * *p* < 0.05, ** *p* < 0.01, *** *p* < 0.001; ns, *p* ≥ 0.05.

To characterize lncRNA-mRNA co-expression patterns within the S100 pathway during *G. parasuis* infection, an in vitro model was established using 3D4/21 cells. Cells were infected at an MOI of 10, and cell viability was monitored from 0 to 24 hpi. A marked reduction in cell viability was observed as early as 6 hpi, with viability declining to 11.84% at 24 hpi (Figure 5A). RT-qPCR analysis showed that pro-inflammatory cytokines *IL6*, *IL8*, *IL11*, and *TNFα* were significantly upregulated at 24 hpi (Figure 5B). Five representative DE mRNAs selected from the lung RNA-seq data were also significantly induced at 24 hpi in 3D4/21 cells, consistent with their expression patterns in lung tissues (Figure 5C).

To validate the co-expression relationships of S100 pathway-related lncRNA-mRNA pairs, time-series RT-qPCR was performed on the two representative lncRNAs selected from the co-expression analysis and their co-expressed mRNAs at 0, 6, 9, 12, and 24 hpi (Figure 5D). *LOC110259349* was persistently upregulated throughout infection, along with its co-expressed mRNAs *LTB4R2* and *MMP1*. By contrast, *LOC110256217* was significantly upregulated only at early stages (6–9 hpi), matching the activation patterns of its co-expressed mRNAs *PTGDR2* and *GHSR*. Pearson correlation analysis confirmed strong positive correlations for all tested pairs.

## 4. Discussion

*G. parasuis* is the primary etiological agent of Glässer’s disease in pigs, and excessive pulmonary inflammation following infection is a major contributor to tissue damage and mortality. Existing transcriptomic studies have largely been confined to binary comparative designs or in vitro/mixed infection models, lacking stratified analyses of different inflammatory grades in vivo [30,36]. In this study, whole-transcriptome sequencing was performed using a two-grade (mild and severe) CD piglet infection model, identifying 299 nominally DE mRNAs and 408 nominally DE lncRNAs in the mild group, which increased to 625 and 1193, respectively, in the severe group. Using this three-group infection model, the number of S100 pathway-associated nominally DE transcripts expanded alongside increasing pulmonary inflammatory lesions. In addition, candidate co-expressed transcriptional signatures linking lncRNAs, S100 and GPCR molecules were identified based on lncRNA-mRNA co-expression networks.

The S100 protein family represents a conserved group of damage-associated molecular patterns (DAMPs), whose members (e.g., S100A8/A9 and S100A12) drive inflammatory signals through a multi-receptor network involving G-protein-coupled receptors (GPCRs), toll-like receptor-4 (TLR4), and the receptor for advanced glycation end-products (RAGE) [37]. S100A8 and S100A9 are upregulated in a time-dependent manner in the lung, spleen, and other tissues following *G. parasuis* infection [38], and S100A8 can activate the NF-κB signaling pathway via TLR4 to induce pro-inflammatory cytokine release [39]. Previous cell-based studies have shown significant enrichment of the S100 pathway in 3D4/21, PK15, and PAVEC cells [36], but a quantitative comparison across different inflammatory grades at the animal level has been lacking. IPA analysis revealed that the S100 pathway was consistently enriched and predicted to be activated as a core inflammatory module in both mild and severe infections. However, its transcriptional involvement expanded markedly with disease severity—from only 8 DE genes enriched in mild infection to 42 genes in severe infection, accompanied by a substantial increase in pathway significance. Thus, S100 pathway activation is a common response to *G. parasuis* infection, but its transcriptional scope expands substantially in severe cases, likely fueling inflammatory cascade amplification.

GPCRs play complex immunomodulatory roles in bacterial infection: some receptors (e.g., the proton sensor GPR68 and the chemokine receptor CXCR2) can be activated by protons or chemokines to initiate pro-inflammatory signaling cascades [40,41]. Others, like the neuropeptide receptor Mrgpra1, exert negative regulatory functions by restraining excessive neutrophil activation [42]. In addition, S100 family members can directly interact with GPCRs to modulate inflammatory responses [37]. However, how GPCRs coordinately participate in S100-mediated pulmonary inflammation remains incompletely understood. Further analysis revealed that GPCRs accounted for nearly half (19/42) of the S100-associated DE genes in severe infection, exhibiting large-scale coordinated upregulation: *LTB4R2* (14.6-fold), *CCR7* (23.4-fold), *ADORA1* (12.5-fold), *PTAFR* (8.6-fold), and *PTGDR2* (approximately 2.5-fold). This coordinated activation suggests a broad transcriptional involvement of the GPCR family in severe inflammation—spanning chemokine receptors (CCR7[43]), lipid mediator receptors (LTB4R2[44], PTAFR [45]), and metabotropic receptors (ADORA1[46]). They cover multiple functional dimensions, including immune cell recruitment, chemotactic signaling, inflammatory mediator release, and metabolism–inflammation crosstalk, suggesting that GPCR involvement in the S100 pathway is more extensive and diverse in severe compared to mild infection. Lei et al. (2024) also identified GPCR members (ACKR3, GPRC5A) participating in inflammatory responses through the S100 pathway in *G. parasuis*-infected 3D4/21, PK15, and PAVEC cells [36], supporting the conserved role of GPCRs in *G. parasuis* inflammatory signaling.

Downstream of the S100-GPCR-associated inflammatory module, four MMPs were coordinately upregulated in the severe group: *MMP12* (6.1-fold), *MMP8* (4.3-fold), *MMP3* (4.0-fold), and *MMP1* (3.6-fold). MMPs are a key family of extracellular matrix (ECM)-degrading enzymes that, during infectious pulmonary inflammation, regulate cytokine activity and immune cell migration through ECM degradation, thereby participating in tissue damage and remodeling [47,48]. In acute lung injury, *MMP-12* increases the number of neutrophils and macrophages in the lungs and upregulates inflammatory cytokines such as *IL-6* and *TNF-α* [49]; *MMP-8* is involved in pulmonary edema and neutrophil accumulation [50]. *MMP-3* plays a significant pro-inflammatory role in pulmonary inflammation, and its knockdown significantly alleviates lung inflammation, injury, and fibrosis [51]. In addition, *MMP1* is also markedly upregulated in chronic lung diseases and closely associated with immune cell infiltration [52]. The reported pro-inflammatory functions of these MMPs align with the upregulation of pro-inflammatory cytokines (*IL6*, *IL8*, *IL11*, *IL1α*, *TNFα*) in the severe group and the aggravated pulmonary pathology observed in this study. To date, transcriptomic studies on *G. parasuis* infection have primarily focused on the S100 family signaling pathway and GPCRs [30,36], with no reports of coordinated MMP family expression during infection. Together with the widespread transcriptional upregulation of GPCRs in the severe group, these results suggest that GPCRs and MMPs represent co-regulated transcriptional modules that may jointly contribute to the broader S100-centered inflammatory response.

The coordinated upregulation of GPCRs and MMPs points to multi-layer amplification of inflammatory signals, prompting the search for candidate lncRNAs that may be associated with the S100-GPCR-MMP transcriptional modules. Consistent with this notion, WGCNA co-expression analysis identified *LOC110256217* and *LOC110259349* as candidate lncRNAs significantly co-expressed with GPCRs and MMPs. Their temporal patterns in vitro were distinctly different: *LOC110256217* was upregulated only transiently at early infection stages, whereas *LOC110259349* was persistently upregulated up to 24 hpi. This temporal division shows interesting parallels with known mechanisms of lncRNA-mediated inflammatory regulation. For example, lincRNA-Cox2 exhibits a similar early-induction pattern in LPS-stimulated macrophages. As an early primary inflammatory gene regulated by NF-κB, it modulates chromatin remodeling through SWI/SNF complex assembly, thereby promoting transcription of late primary inflammatory genes [53,54]. At the mechanistic level, some lncRNAs have been reported to regulate GPCR signaling. In the brain tissues of preterm mice with inflammation, differentially expressed lncRNAs were significantly enriched in the GPCR signaling pathway [55]. In human cell studies, lncRNA NEAT1 was identified as a key regulator that coordinately modulates multiple GPCRs, including HTR2A and ADRB2 [56]. LINK-A mediates crosstalk between GPCR and PIP3 signaling pathways to regulate downstream signals [57]. Regarding MMP regulation, studies have demonstrated that lncRNAs can affect MMP expression through various mechanisms. For instance, lncRNA RP11-297P16.4 targets *MMP-2/9* through a ceRNA mechanism [58]. lncRNA OSTM1-AS1 upregulates *MMP-9* expression by sponging miR-491-5p [59]. These known mechanisms provide clues for understanding the potential functions of the lncRNAs identified in this study, although their specific regulatory networks in *G. parasuis* infection require further investigation.

**Figure 6:**
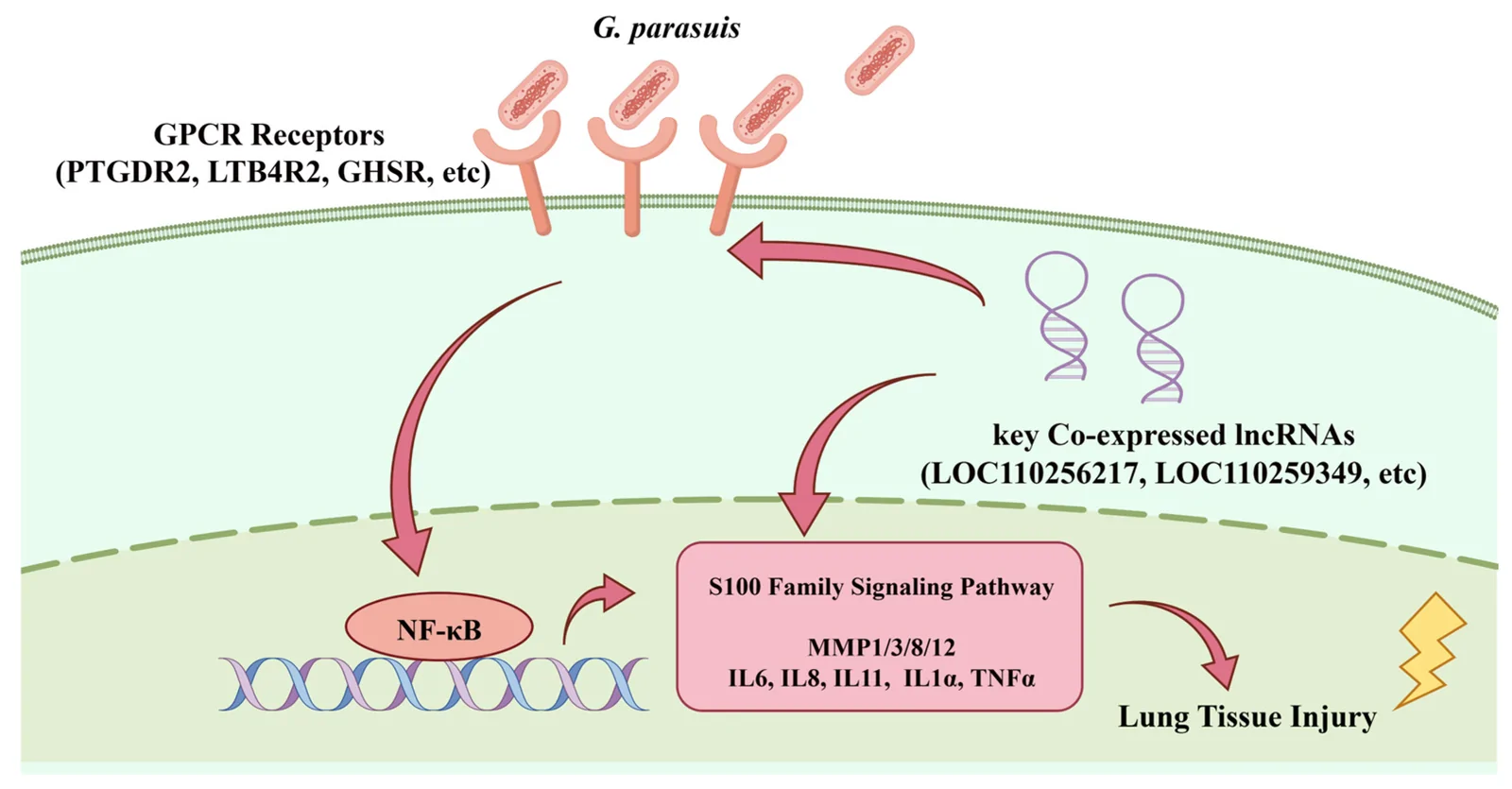
Proposed model of lncRNA-mRNA interaction networks within the S100 signaling pathway during *G. parasuis* infection. Arrows represent hypothesized transcriptional associations derived from co-expression analysis and in-silico IPA prediction.

Integrating the in vivo and in vitro findings, a putative working model (Figure 6) was constructed. In this model, transcriptional upregulation of GPCR components (e.g., *LTB4R2*, *GHSR*, *PTGDR2*), MMPs (e.g., *MMP1/3/8/12*), and pro-inflammatory cytokines (*IL6*, *IL8*, *IL1α*, *IL11*, *TNFα*) is associated with the inflammatory response during *G. parasuis* infection. Moreover, *LOC110256217* and *LOC110259349* exhibit distinct temporal transcript profiles correlated with early- and late-phase inflammatory responses, suggesting they may be differentially associated with inflammatory processes. This model is hypothesis-generating and requires functional validation (e.g., knockdown/overexpression experiments) to establish direct regulatory relationships, as WGCNA reflects only co-expression correlation. Although the use of uncorrected raw *p*-values (no FDR correction) may increase false positives, candidate discovery was prioritized in this exploratory small-sample study, and key nominally DE transcripts were independently validated by RT-qPCR. The sample size of three pigs per group is limited by the CD piglet model, and larger cohorts would help validate the statistical reliability of the graded transcriptional features. The severe cytotoxicity at 24 hpi (cell viability 11.84%) may confound transcriptional changes at this late time point. Thus, the 24 hpi data warrant cautious interpretation, whereas the earlier time points with relatively preserved viability provide more reliable co-expression evidence. The 3D4/21 model serves only as complementary support for in vivo-derived trends, not as mechanistic proof. Additionally, the 3D4/21 cell culture cannot recapitulate the multicellular microenvironment of lung tissue, and the in vitro temporal patterns require further in vivo validation. Furthermore, the applicability of these findings to commercial pig farms requires further verification under field conditions. Nevertheless, the transcriptomic atlas and lncRNA-mRNA interaction pairs generated in this study provide a reliable candidate resource for future functional experiments.

## 5. Conclusions

Whole-transcriptome profiling was performed on lung tissues from healthy, mild, and severe serotype 5 *G. parasuis*-infected CD piglets. Nominally DE mRNAs and lncRNAs were identified that exhibited graded changes with inflammation severity in both the Mild vs. Control and Severe vs. Control comparisons, with substantially more transcripts differentially expressed in the severe than in the mild group. IPA analysis confirmed that the S100 pathway serves as a core module of graded pulmonary inflammation, with GPCRs such as *PTGDR2*, *LTB4R2*, and *GHSR* being prominently co-expressed within the S100-associated inflammatory network. WGCNA co-expression network analysis identified candidate lncRNAs including *LOC110256217* and *LOC110259349*, and in vitro temporal infection models revealed their distinct temporal co-expression patterns, suggesting potential differential involvement at early and late inflammatory stages. Collectively, these findings propose a putative lncRNA-S100-GPCR-associated inflammatory module in serotype 5 *G. parasuis* infection, offer candidate transcripts for future functional validation and investigation into host resilience, and provide a transcriptomic resource and candidate lncRNA-mRNA interaction pairs for elucidating the molecular mechanisms of graded lung injury. Notably, the limited animal sample size restricts the statistical robustness of these graded inflammatory findings.

## Data Availability

The raw sequence data reported in this paper have been deposited in the Genome Sequence Archive at the National Genomics Data Center, China National Center for Bioinformation/Beijing Institute of Genomics, Chinese Academy of Sciences, under BioProject accession number PRJCA071673, and will be publicly released upon publication at https://ngdc.cncb.ac.cn/gsa.

## Supplementary Materials

The following supporting information can be downloaded at: https://www.mdpi.com/article/10.3390/ani16172645/s1, Figure S1: PCA of mRNA expression profiles from control, mild, and severe groups. Figure S2: Top 20 enriched IPA disease and function annotations of nominally DE mRNAs. (A) Mild vs. Control; (B) Severe vs. Control. Figure S3: IPA regulatory network of the S100 family signaling pathway (severe vs. control). Figure S4: Characterization of lncRNA expression profiles. (A) Counts of known and novel lncRNAs detected per sample. (B,C) Transcript length and exon number distributions of known lncRNAs, novel lncRNAs, and mRNAs. (D) Box plots of lncRNA FPKM values across the Control (red), Mild (green), and Severe (blue) groups. (E) Density distribution of lncRNA FPKM values. (F) PCA of lncRNA expression profiles across groups. Figure S5: WGCNA co-expression network heatmaps of nominally DE lncRNAs and their co-expressed mRNAs. (A,B) Heatmaps constructed for the Mild vs. Control (A) and Severe vs. Control (B) comparisons (soft-thresholding power = 7). Axes contain all WGCNA input transcripts. Darker red denotes higher co-expression weight; side color bars label distinct co-expression modules. Table S1. Individual clinical symptom scores for CD piglets after *Glaesserella parasuis* challenge. Raw scoring data were derived from reference [34] (Liu et al., 2022, published in Chinese). Table S2: Primers information for the mRNAs used for RT-qPCR. Table S3: Summary of RNA-seq quality control and mapping statistics for lung tissue samples from control, mild, and severe groups. Table S4: Nominally DE mRNAs were identified between the mild and control groups. Table S5: Nominally DE mRNAs were identified between the severe and control groups. Table S6: Enriched Ingenuity canonical pathways in the mild group compared with the control group. Table S7: Enriched Ingenuity canonical pathways in the severe group compared with the control group. Table S8: Enriched diseases and function annotations in mild group versus control group. Table S9: Enriched diseases and function annotations in the severe group compared with the control group. Table S10: Nominally DE lncRNAs in the mild group compared with the control group. Table S11: Nominally DE lncRNAs in the severe group compared with the control group. Table S12: Detailed information of co-expression pairs between nominally DE lncRNAs and nominally DE mRNAs in the Mild vs. Control comparison. Table S13: Detailed information of co-expression pairs between nominally DE lncRNAs and nominally DE mRNAs in the Severe vs. Control comparison. Table S14: Genomic features of LOC110256217 and LOC110259349 in the porcine reference genome.

## Abbreviations

The following abbreviations are used in this manuscript:

CD	colostrum-deprived
DAMP	damage-associated molecular pattern
DE	differentially expressed
ECM	extracellular matrix
FC	fold change
GPCR	G-protein-coupled receptor
hpi	hours post-infection
IPA	Ingenuity Pathway Analysis
lncRNA	long non-coding RNA
LOS	lipooligosaccharide
MMP	matrix metalloproteinase
PCA	principal component analysis
RAGE	receptor for advanced glycation end-products
RT-qPCR	reverse transcription quantitative real-time PCR
TOM	topological overlap matrix
WGCNA	weighted gene co-expression network analysis
